# Supplementary material for: Accessing MHz Operation at 2 V with Field‐Effect Transistors Based on Printed Polymers on Plastic
Source: Adv Sci (Weinh). 2018 Dec 14;6(4):1801566. doi: 10.1002/advs.201801566 (PMC6382309; doi:10.1002/advs.201801566)
Supplement: Supplementary file 1 — Supplementary [file ADVS-6-1801566-s001.pdf]

Copyright WILEY-VCH Verlag GmbH & Co. KGaA, 69469 Weinheim, Germany, 2018.

ADVANCED SCIENCE Open Access

Supporting Information

for *Adv. Sci.,* DOI: 10.1002/advs.201801566

Accessing MHz Operation at 2 V with Field-Effect Transistors Based on Printed Polymers on Plastic

*Andrea Perinot and Mario Caironi**

Copyright WILEY-VCH Verlag GmbH & Co. KGaA, 69469 Weinheim, Germany, 2016.

Supporting Information

Accessing MHz Operation at 2 V with Field-Effect Transistors Based on Printed Polymers on Plastic

Andrea Perinot, Mario Caironi*

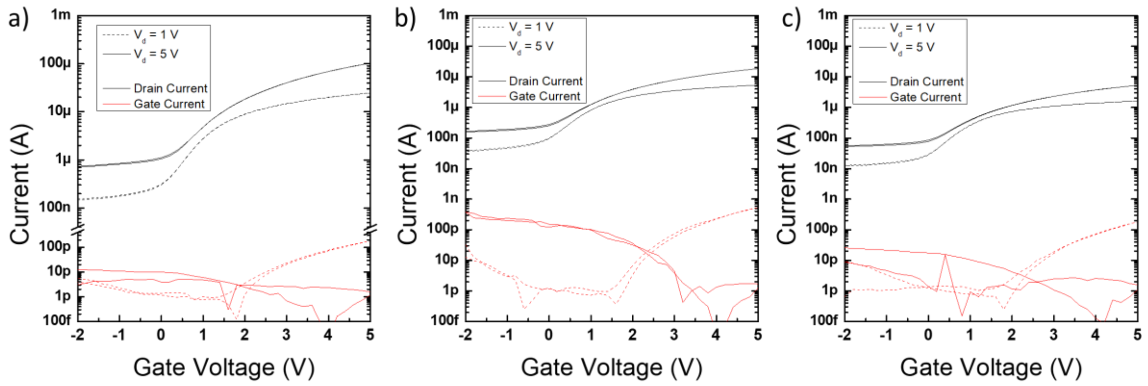

Figure S1. Transfer curves for devices with a) L = 1.5 µm, b) L = 5.5 µm, c) L = 17.5 µm.

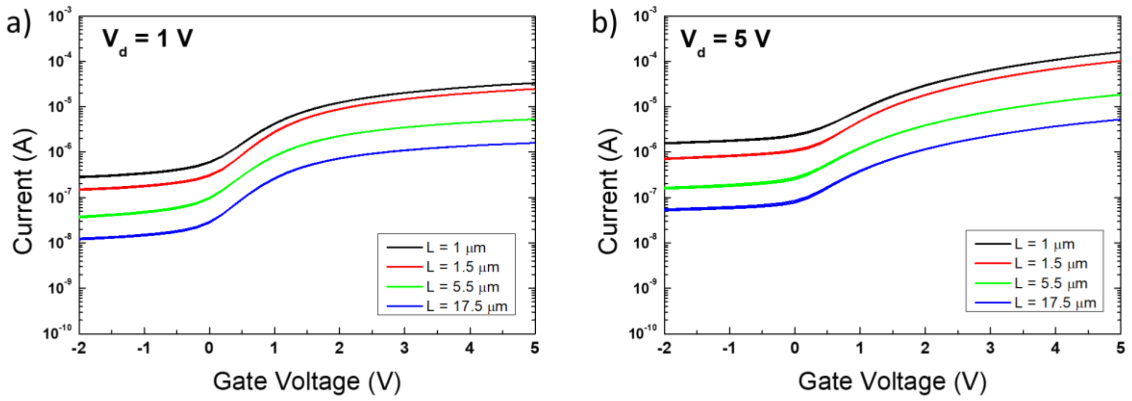

Figure S2. Transfer curves for the FETs with all the realized channel lengths: a) linear regime and b) saturation regime.

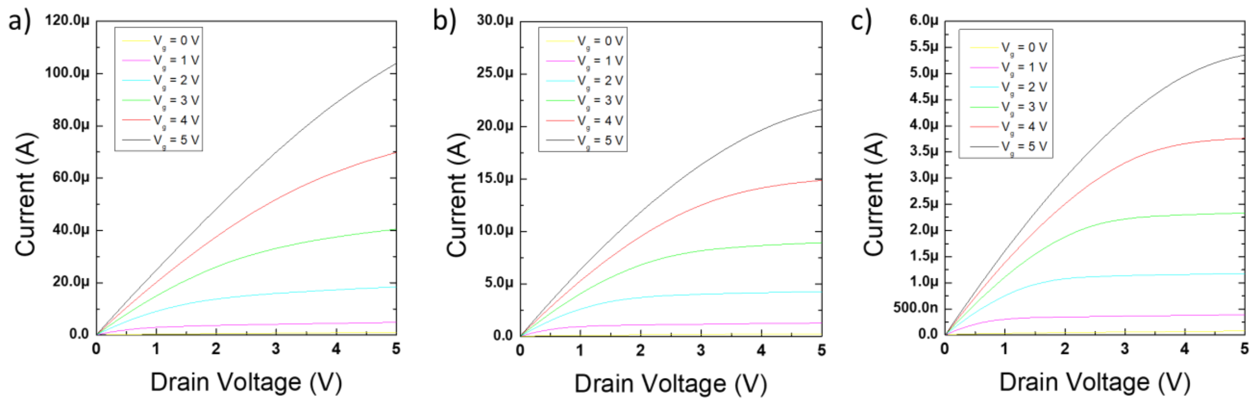

Figure S3. Output curves for devices with a) L = 1.5 µm, b) L = 5.5 µm, c) L = 17.5 µm.

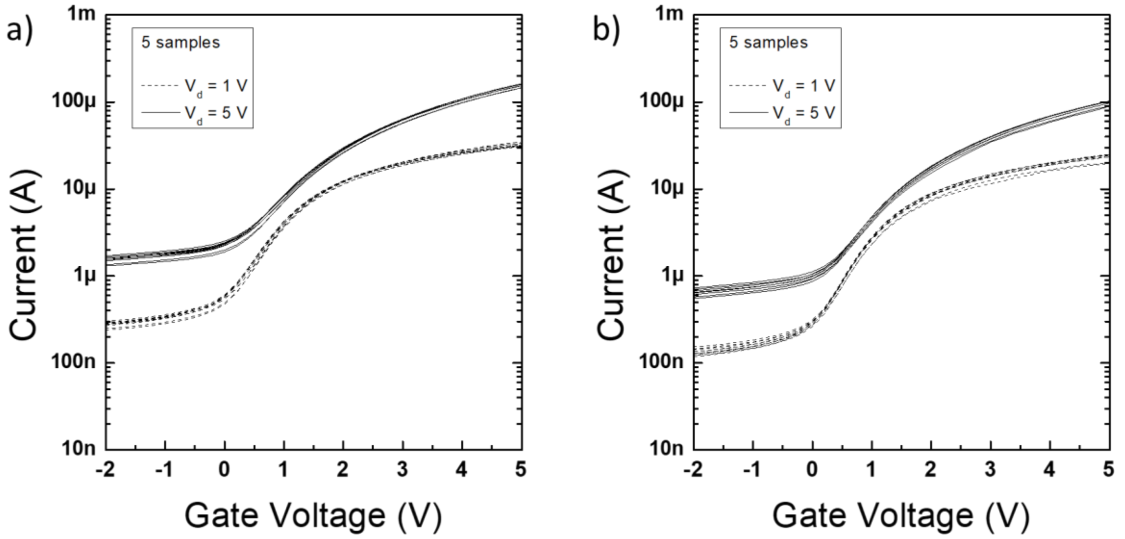

Figure S4. Superimposed transfer curves for 5 devices with a) L = 1 μm, b) L = 1.5 μm.

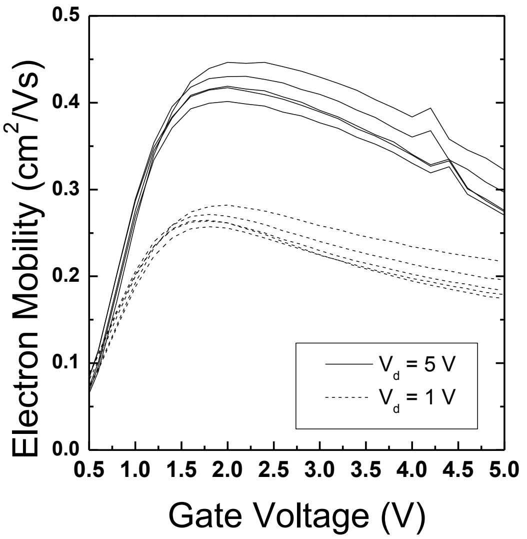

Figure S5. Superimposed apparent charge mobility curves for 5 devices with L = 1 μm.

**Parameter [Unit]**	**Mean**	**Standard Deviation**
Mobility ($V_g = V_d = 5$ V) [cm^2/Vs]	0.287	0.022
μ_0 [cm^2/Vs] *	0.291	0.004
γ *	0.052	0.019
Threshold Voltage (V) *	0.559	0.028

a) **Extracted with Differential Method[1]**

Table S1. Mean and standard deviation of selected figures of merit for the devices of Figure S5.

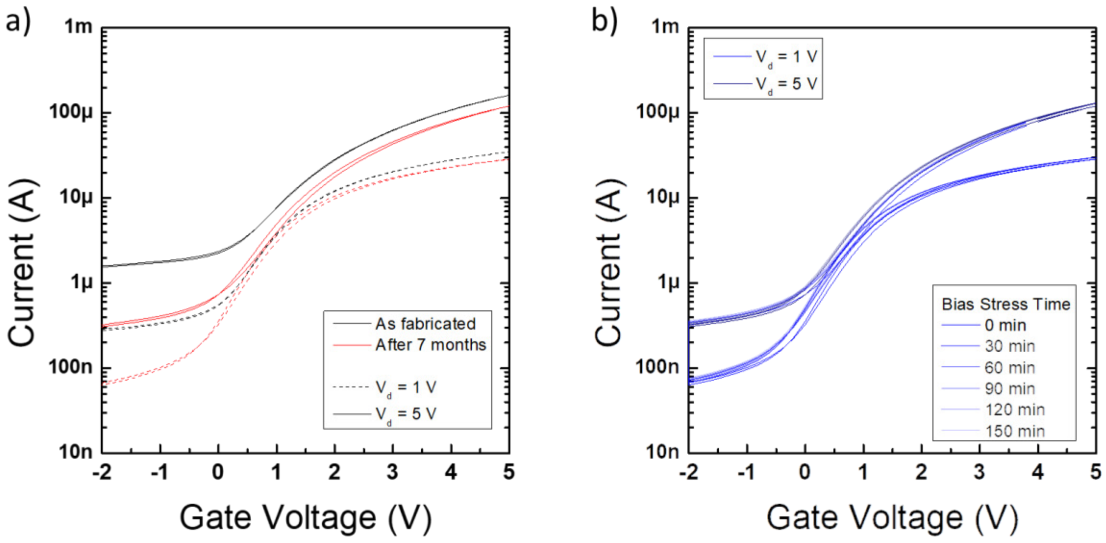

Figure S6. a) Measured transfer curves for a device with L = 1 μm before and after storage in nitrogen atmosphere for 7 months, b) measured transfer curves for the same device upon operational stress for 150 minutes (curves measured every 30 minutes).

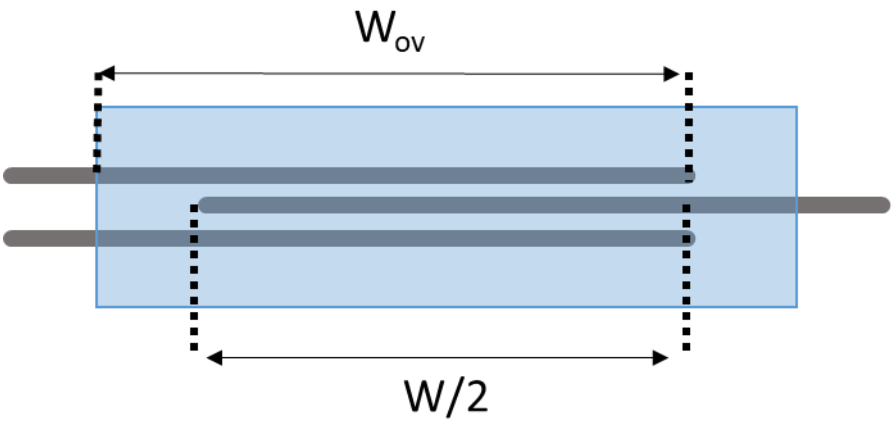

Figure S7. Sketch of the layout of an FET device of this work, graphically illustrating the definition of W and W_{ov}.

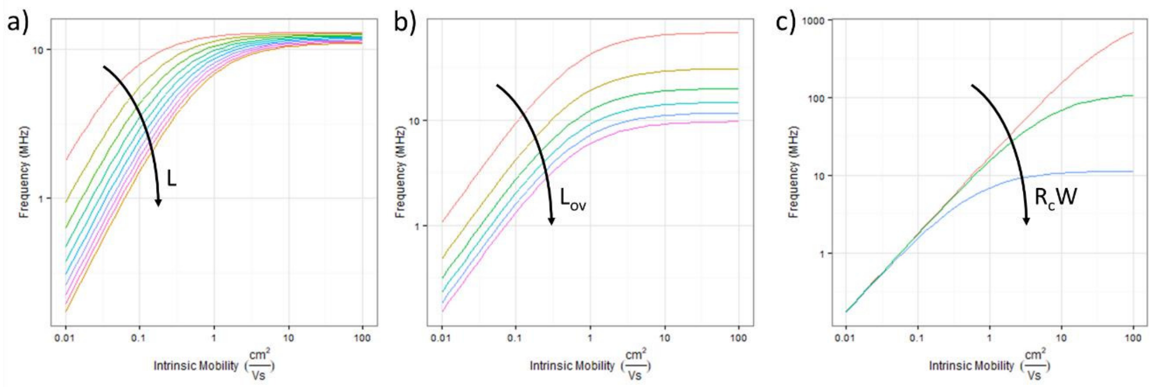

Figure S8. Theoretical estimation of the FETs transition frequency versus intrinsic mobility of the semiconductor, according to the model proposed by Klauk [2]. Trends with respect to the variation of: a) channel length L from 0.1 to 1 µm, 0.1 µm step; b) overlap length L_{ov} from 0 to 2 µm, 0.4 µm step; c) width-normalized contact resistance R_CW from 10 to 1000 Ωcm, one point per decade. Calculations parameters (when not varying): L = 1 µm, R_CW = 1015 Ωcm, C_{diel} = 39 nF/cm^2, V_g = 5 V, V_{th} = 0.5 V, V_d = 1 V.

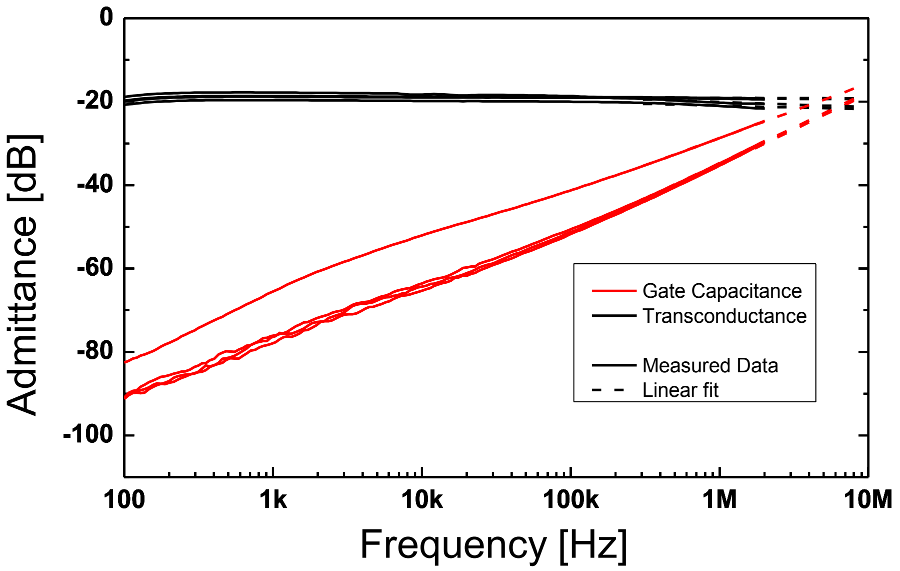

Figure S9. Superimposed curves of the measured transconductance and gate capacitance for 4 devices with L = 1 µm and a bias voltage of 5 V. Devices were measured after 7 months from fabrication.

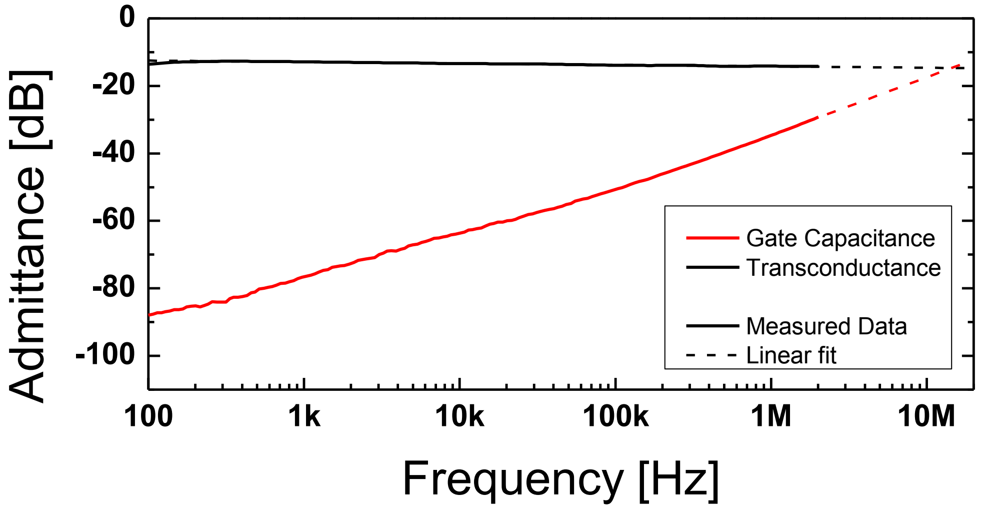

Figure S10. Measured curves of the transconductance and gate capacitance for the best device with L = 1 μm and a bias voltage of 7 V, yielding f_t = 14.4 MHz. Device was measured after 7 months from fabrication.

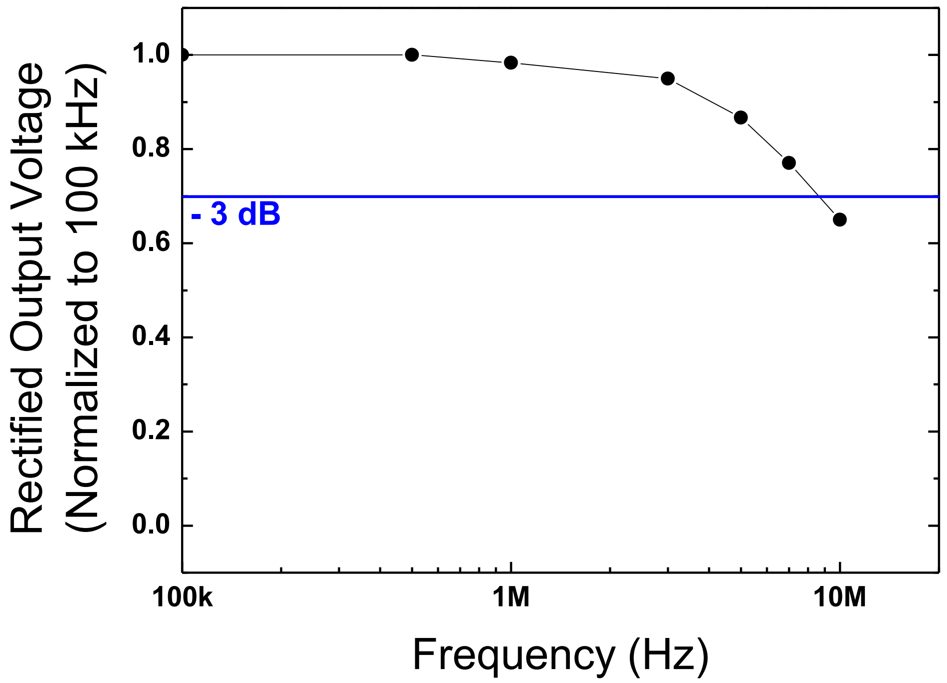

Figure S11. Measured output voltage of the realized rectifier versus input voltage frequency, for an input signal amplitude of 8 V.

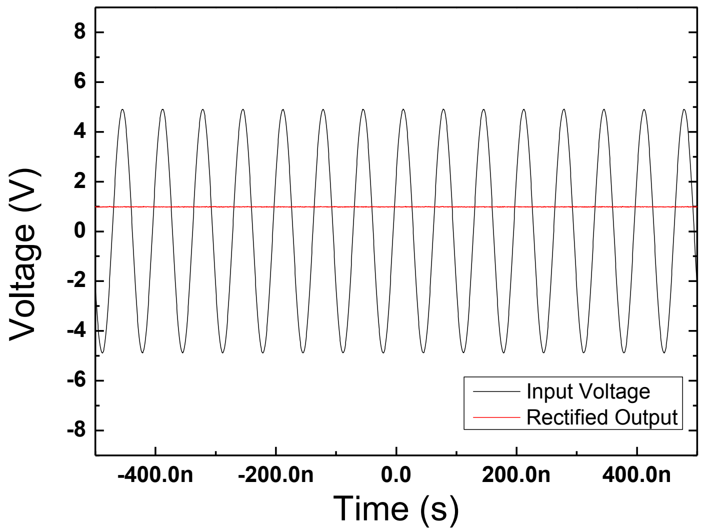

Figure S12. Input and output voltage waveforms for the realized rectifier at an input voltage frequency of 15 MHz and an input voltage amplitude of 5 V.

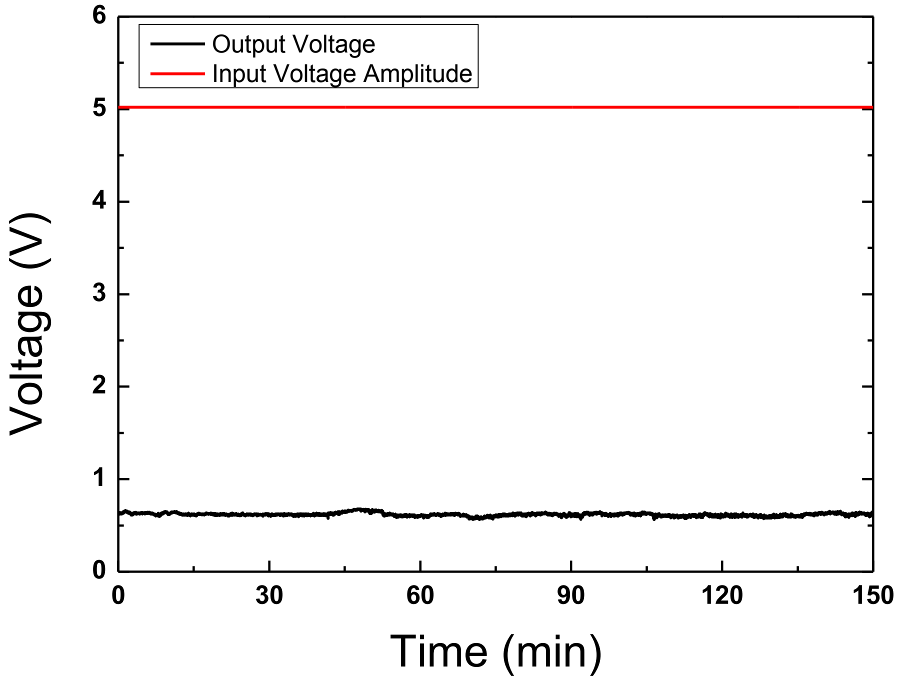

Figure S13. Time-dependent measurement of output voltage for the realized rectifier at an input voltage frequency of 15 MHz and an input voltage amplitude of 5 V, upon constant operation for 150 minutes.

Modeling of the DC output voltage of the FET rectifier and its relationship vs. frequency.

To calculate the output voltage V_{dc} of the rectifier (see Figure SD1) we make the following assumptions: 1) V_{dc} is constant; 2) the smoothing capacitor is fully charged at V_{dc} at the time of this analysis; 3) the FET threshold voltage is 0 V. The following mathematical relationship is derived by considering that Q_{FET}, the charge driven from the supply to the output when the FET is ON (during one cycle), must compensate for the following:

- Q_{RL}: the amount of charge that is discharged from the output due to the load resistor;
- Q_{RR}: the amount of charge that is discharged from the output due to the reverse current of the FET;
- Q_C: the amount of charge that is needed to charge the FET channel capacitance.

We remark that the assumption that Q_C is fully charged by part of Q_{FET} at each cycle is largely simplifying the physical behavior of the device. More elaborate models for the transient behavior of FETs can be found for example in [3].

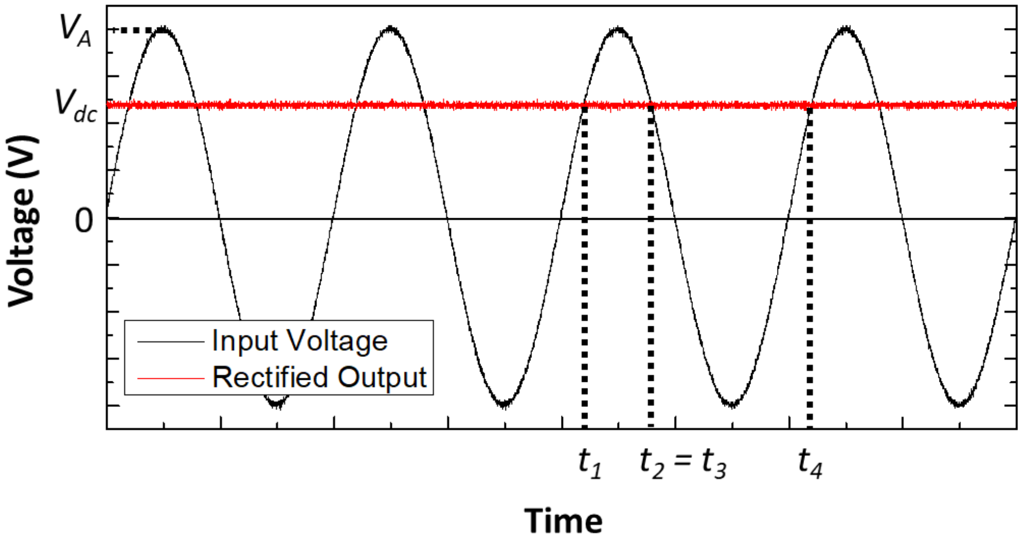

Figure SD1. Graphical identification of the parameters defined in our model for the DC output voltage of the FET rectifier.

We define the following quantities:

- R_L : the load resistance of the rectifying circuit;
- $R_{R,W}$: the resistance of the FET in the reverse bias regime (normalized per unit W);
- V_A : the amplitude of the input oscillating voltage;
- W : the channel width of the FET;
- $I_{r,FET} = -(V_A \sin \omega t - V_{DC})W/R_{R,W}$: the reverse current of the FET;
- L : the channel length of the FET;
- ε_0, ε_r : dielectric permittivity of vacuum and relative dielectric constant of the insulator;
- t_d : thickness of the FET insulator layer;
- ω : the angular frequency of the input oscillating voltage.

The individual charge contributions can be expressed as follows:

$$Q_{RL} = \frac{V_{DC}}{R_L}\frac{2\pi}{\omega}$$

$$Q_{RR} = \int_{t_3}^{t_4} I_{r,FET} dt = \frac{2V_A W}{R_{R,W}\omega}\sqrt{1-\left(\frac{V_{DC}}{V_A}\right)^2} + \frac{2V_{DC} W}{R_{R,W}\omega}(\pi - \cos^{-1}\frac{V_{DC}}{V_A})$$

$$Q_C = \frac{\varepsilon_0\varepsilon_r\left(W\frac{2}{3}L\right)}{t_d}(V_A - V_{DC})$$

$$Q_{FET} = \frac{\mu\varepsilon_0\varepsilon_r W}{2t_d L}\int_{t_1}^{t_2}(V_A \sin\omega t - V_{DC})^2\, dt$$

$$= \frac{\mu\varepsilon_0\varepsilon_r W}{2t_d L\omega}\left[(V_A^2 + 2V_{DC}^2)\cos^{-1}\frac{V_{DC}}{V_A} - 3V_A V_{DC}\sqrt{1-\left(\frac{V_{DC}}{V_A}\right)^2}\right]$$

Where:

$$t_1 = \frac{1}{\omega}\sin^{-1}\frac{V_{DC}}{V_A}, \quad t_2 = \frac{1}{\omega}(\pi - \sin^{-1}\frac{V_{DC}}{V_A}), \quad t_3 = t_2, \quad t_4 = \frac{1}{\omega}(2\pi + \sin^{-1}\frac{V_{DC}}{V_A})$$

Substitution of these quantities in $Q_{RL} + Q_{RR} + Q_C = Q_{FET}$, by additionally defining $x = V_{DC}/V_A$ yields:

$$\frac{2\pi}{R_L}x + \frac{2W}{R_{R,W}}\sqrt{1-x^2} + \frac{2W}{R_{R,W}}x(\pi - \cos^{-1}x) + \frac{\varepsilon_0\varepsilon_r\left(W\frac{2}{3}L\right)}{t_d}(1-x)\omega$$
$$= \frac{\mu\varepsilon_0\varepsilon_r W}{2t_d L}V_A\left[(1+2x^2)\cos^{-1}x - 3x\sqrt{1-x^2}\right]$$

DC output voltage of the FET rectifier at low frequency (limit ω = 0)

We calculated the behavior of the formula derived above for ω = 0 as a function of some parameters, in order to determine how to improve the ratio $x = V_{DC}/V_A$. The values of the fixed parameters are as follows:

- $R_{R,W} = 0.24502\ M\Omega cm$
- $R_L = 1\ M\Omega$
- $\mu = 0.45\ cm^2/Vs$
- $V_A = 8\ V$
- $L = 1\ \mu m$
- $C_{diel} = 39\ nF/cm^2$
- $W = 800\ \mu m$

We found that x increases steadily with the increase of $R_{R,W}$ and R_L as expected (Fig SD2), since we are reducing the amount of current discharged per cycle from the capacitance. Indeed, if only one of the two is reduced, V_{DC}/V_A reaches a plateau at a certain value and cannot be improved further. The reduction of both resistances at the same time is needed to achieve a high x ratio (Fig SD3).

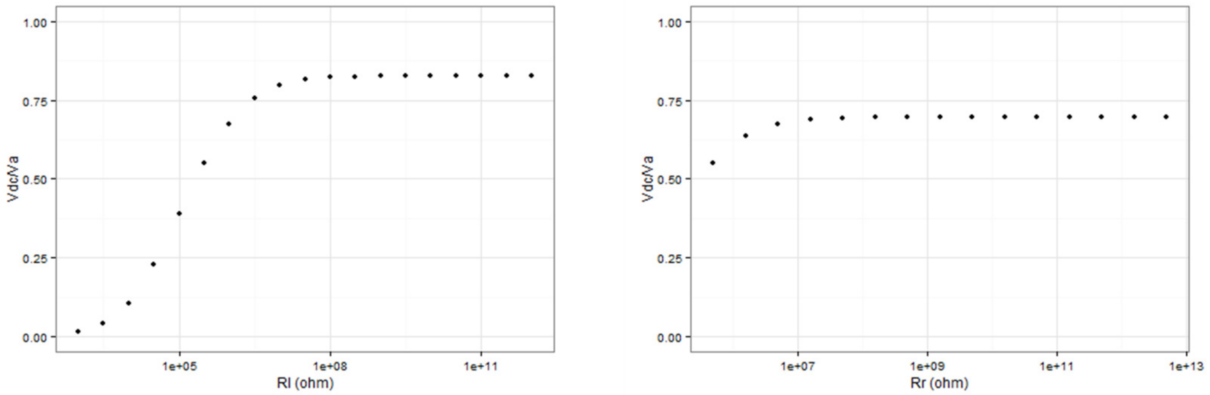

Figure SD2. Calculation of the variation of the ratio V_{DC}/V_A for varying $R_{R,W}$ (right) and R_L (left).

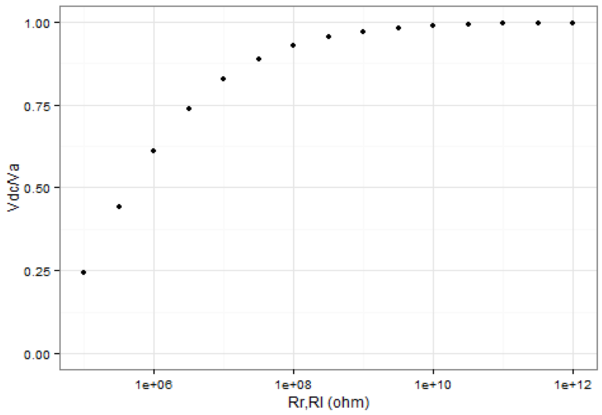

Figure SD3. Calculation of the variation of the ratio V_{DC}/V_A for varying $R_{R,W} = R_L$.

In real applications, usually, R_L is set and the designer can operate on the transistor dimension W to achieve the desired performance. Unfortunately, increasing W not only increases the transistor ON current but also decreases $R_{R,W}$, so that this approach does not help after the plateau for x is reached. Once R_L is set, and W has been maximized, it is paramount to appropriately increase $R_{R,W}$ (Fig SD4).

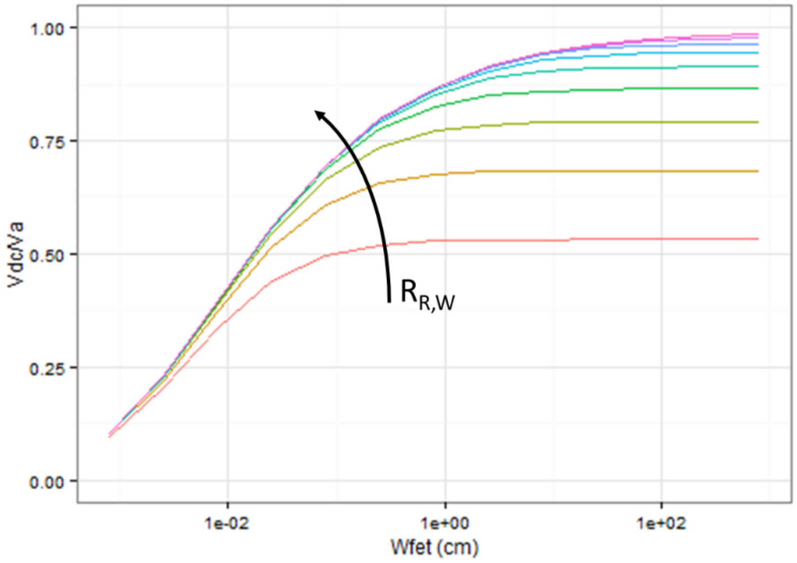

Figure SD4: Calculation of the variation of the ratio V_{DC}/V_A for varying W and $R_{R,W}$ varying in the interval 0.24×10^5 to 0.24×10^9 Ωcm, 0.5 decades per step.

Calculation of the output voltage of the FET rectifier vs. frequency

The maximum speed of the rectifier is limited by the fact that a fixed charge must be provided to the transistor at each OFF/ON transition before it can drive current to the output. We can calculate V_{DC}/V_A vs. frequency $f = \omega/2\pi$ for different design parameters. We set R_L = 1 MΩ, V_A = 5 V and μ = 0.3 cm^2/Vs.
If we set $R_{R,W} = 0.24502\ M\Omega cm$, which is the actual value for our devices, and we vary the transistor width W_{FET} we have that we are only improving the ratio x, while there is no influence in the maximum operational frequency (Fig SD5). This is in agreement with the basic consideration that W does not influence the f_t of the FET.

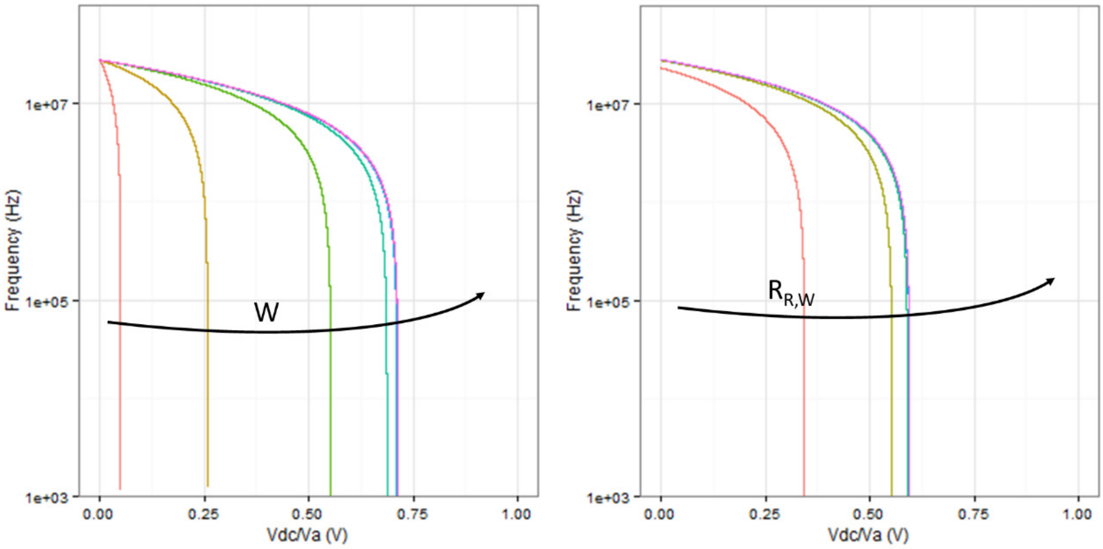

Figure SD5. Calculation of V_{DC}/V_A vs. frequency for: 1) varying W in the range 800×10^{-6} cm to 800 cm, 1 decade per step (left panel); 2) varying $R_{R,W}$ in the range 0.24×10^5 to 0.24×10^9 Ωcm, 1 decade per step (right panel).

The same can be observed by setting W to 800 μm and varying $R_{R,W}$, with no influence on the maximum operational frequency (after reaching the plateau for V_{DC}/V_A).
This illustrates that we can design properly a rectifier capable of supplying the desired power to the output with constant V_{DC} without introducing limitation to the maximum operational frequency.
When we compare these calculations with our results, we observe that the ratio between the output voltage and the amplitude of the input is predicted to be 0.55, while our data yields a ratio of 0.46. The small discrepancy can be attributed to the fact that the FET the mobility of our FET is gate- and drain-voltage dependent. Finally, we observe a good agreement between the measured and calculated frequency behavior of the fabricated rectifier (Fig SD6).
Despite the first-order assumptions we adopted in our model, our model allows the identification of the parameters influencing the DC output voltage and the maximum operational frequency of a rectifier based on a transdiode FET, providing the guidelines for the correct design and integration of such a circuit into RF applications.

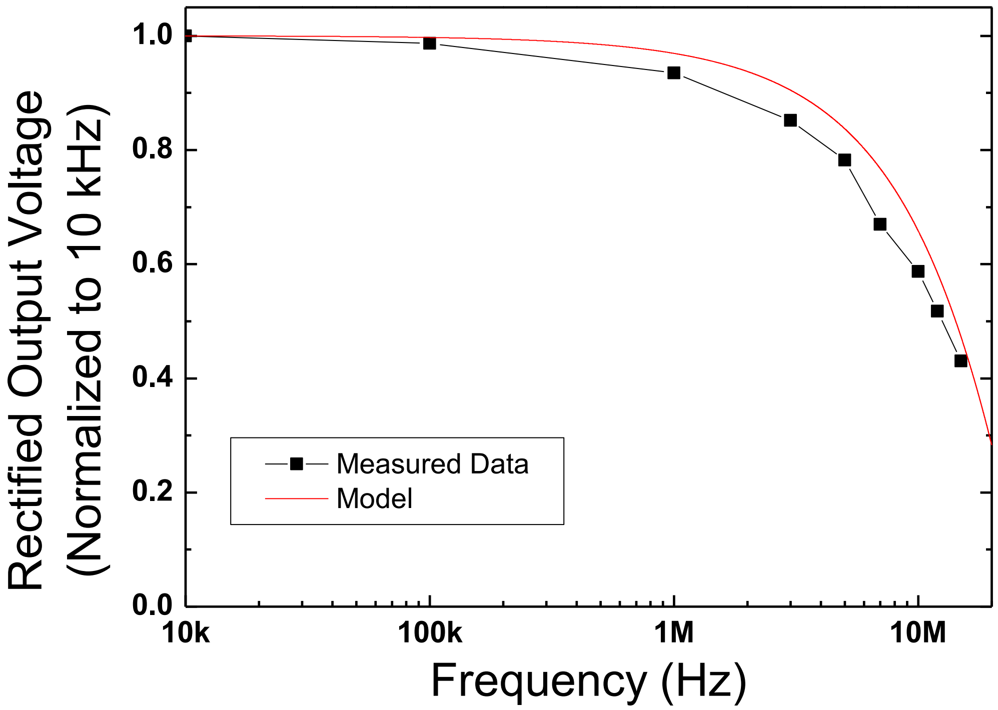

Figure SD6. Measured and calculated curve for the rectified output (normalized to 10 kHz) versus input voltage frequency at 5 V.

[1] D. Natali, L. Fumagalli, M. Sampietro, J. Appl. Phys. **2007**, 101, 014501.
[2] H. Klauk, Adv Electron Mater **2018**, 0, 1700474.
[3] Y. Tsividis, C. McAndrew, Operation and Modeling of the MOS Transistor, Oxford Univ. Press, 2011.
